# Source localisation and its uncertainty quantification after the third DPRK nuclear test

**DOI:** 10.1038/s41598-018-28403-z

**Published:** 2018-07-05

**Authors:** Pieter De Meutter, Johan Camps, Andy Delcloo, Piet Termonia

**Affiliations:** 1 Belgian Nuclear Research Institute, Mol, 2400 Belgium; 2 Royal Meteorological Institute of Belgium, Brussels, 1180 Belgium; 3 Ghent University, Department of Physics and Astronomy, Ghent, 9000 Belgium

## Abstract

The International Monitoring System is being set up aiming to detect violations of the Comprehensive Nuclear-Test-Ban Treaty. Suspicious radioxenon detections were made by the International Monitoring System after the third announced nuclear test conducted by the Democratic People’s Republic of Korea (DPRK). In this paper, inverse atmospheric transport and dispersion modelling was applied to these detections, to determine the source location, the release term and its associated uncertainties. The DPRK nuclear test site was found to be a likely source location, though a second likely source region in East Asia was found by the inverse modelling, partly due to the radioxenon background from civilian sources. Therefore, techniques to indirectly assess the influence of the radioxenon background are suggested. In case of suspicious radioxenon detections after a man-made explosion, atmospheric transport and dispersion modelling is a powerful tool for assessing whether the explosion could have been nuclear or not.

## Introduction

The Comprehensive Nuclear-Test-Ban Treaty (CTBT) was opened for signature in 1996. Once the Treaty enters into force, it will ban atmospheric, underwater and underground nuclear explosions. The International Monitoring System is almost fully operational and already measures infrasound, hydroacoustic and seismic waves, together with certain airborne radionuclides. The radionuclide measurement system will consist of eighty ground stations measuring radioactive particulates. Forty of these stations will also be equipped with noble gas detectors, allowing the measurement of airborne concentrations of ^131*m*^Xe, ^133*m*^Xe, ^133^Xe and ^135^Xe (with half-lives of respectively 11.84 d, 2.20 d, 5.25 d and 9.14 h^1^). These radioactive xenon isotopes (hereafter radioxenon) are selected for monitoring due to their significant production after a nuclear explosion, their half-life and their likelihood to seep through the ground in case of an underground nuclear explosion. Observations from the International Monitoring System are made available to member states that have signed the CTBT in order to verify compliance with the Treaty.

In the past, the Democratic People’s Republic of Korea (DPRK) announced several times to have conducted a nuclear test. Although the International Monitoring System has not yet been fully installed (90% to date^2^), these tests provide interesting cases for testing the capability to detect clandestine nuclear tests and to confirm announced nuclear tests. Seismic waves were detected after each DPRK nuclear test^2^. These observations allow the determination of the timing, location and magnitude of the explosion. In order to discriminate conventional explosions from nuclear explosions, a specific signature of radionuclides needs to be determined. If measured, atmospheric transport models can be used to verify whether these radionuclides originated from the nuclear test site. This task is complicated by two factors: (i) the DPRK nuclear tests were conducted underground, so that it is a priori not sure if and what fraction of radionuclides were released into the atmosphere; (ii) there is an ongoing background of radioxenon from civilian sources such as nuclear power plants and medical isotope production facilities, resulting in frequent detections by the International Monitoring System^3^.

The inverse problem in atmospheric transport and dispersion modelling (ATM) consists of finding source parameters such as release location, release height, release amount and release period, based on a set of observations. The (dis)agreement between these observations and their corresponding simulated values obtained from the selected source parameters, is quantified by a cost function or likelihood function. An overview of different inverse modelling methods has recently been published^4^. Examples of inverse atmospheric transport modelling of point sources include the calculation of the release amount of radionuclides from nuclear accidents, such as the Fukushima Dai-ichi nuclear power plant accident in 2011^5–8^, or modelling the release of ash from volcano eruptions like the Eyjafjallajökull in 2010^9,10^ which severely disturbed air traffic above Europe. For these cases, inverse modelling is often followed by forward modelling using the best source parameters obtained from the inverse modelling, in order to know the concentration of a tracer at any location and any time. This complements the observations, which are typically sparse. In other cases, inverse modelling is used to locate possible sources (such as the source location of ^131^I detections made in Europe in 2011^11^). Inverse atmospheric transport modelling is also used in other areas of research, such as the quantification of greenhouse gas fluxes in the context of climate science.

In the past, radioxenon observations were linked to nuclear tests conducted by the DPRK^12–14^. Of particular interest are the specific combinations of ^131*m*^Xe and ^133^Xe that were detected seven to eight weeks after the third announced nuclear test in February 2013^14^. These detections were made in three samples taken at the station RN38 in Takasaki (Japan) between 7–9 April 2013 and in two samples taken at the station RN58 in Ussuriysk (Russia) between 12–13 April 2013. The radioxenon detections were attributed to a nuclear explosion, mainly based on their unusual occurrence compared to the station’s record^14^. Atmospheric transport and dispersion modelling was used to calculate the source-receptor-sensitivities between the stations (or “receptors”) and possible sources. By calculating correlations between the source-receptor-sensitivities and the observed concentrations, so-called “Possible Sources Regions”^15^ were constructed. The nuclear test site Punggye-ri was found to fall within these possible source regions^14^.

More complex ATM methods for assessing the third nuclear test conducted by the DPRK have been made using Bayesian methods to find source parameters^16,17^, and using an optimisation method to find the possible source regions^18^. All three studies identified the Punggye-ri nuclear test site as a likely source of the suspicious radioxenon detections. An automated methodology for principled computational inference for Bayesian source reconstruction was described and applied to the third nuclear test conducted by the DPRK^17^. In another study, a method for fusing information from the radionuclide verification regime with the seismic verification regime was proposed^18^, which method consists of assessing the cost function values at only those locations where seismic activity had been measured.

After the announced nuclear test in January 2016, elevated ^133^Xe concentrations were measured by the International Monitoring System, compatible with a delayed release from the Punggye-ri nuclear test site^19^. However, due to the lack of detections of other radioxenon isotopes, it could not be excluded that the observed ^133^Xe originated from civilian sources. In such cases, ATM is able to narrow down possible source regions and provide information on the release period and release amount. The resulting area, preferably as small as possible, depends on the meteorological conditions and the network configuration: more observations at different geotemporal locations will result in a smaller area^20^.

In this paper, the existing methodology^19^ was extended to improve the estimation of the source location and to address the problem of the radioxenon background. Inverse long-range atmospheric transport and dispersion modelling was used to assess the source location of the radioxenon detections previously described^14^. The following two questions were addressed in particular: (i) how precise can we narrow down possible source regions and (ii) can these detections be linked to a delayed release from the Punggye-ri nuclear test site. An uncertainty quantification was provided by using unique meteorological ensemble data, which allowed to construct grid box probability maps for the source localisation.

## Source localisation of the Xe-133 detections

In this section, the possible source locations of the selected ^133^Xe samples will be determined. First, inverse modelling was applied over the northern hemisphere using deterministic weather data (Methods). From that assessment, a smaller region of interest was identified for which the inverse modelling was repeated using the ensemble weather data (Methods).

### Assessment over the northern hemisphere

**Figure 1:**
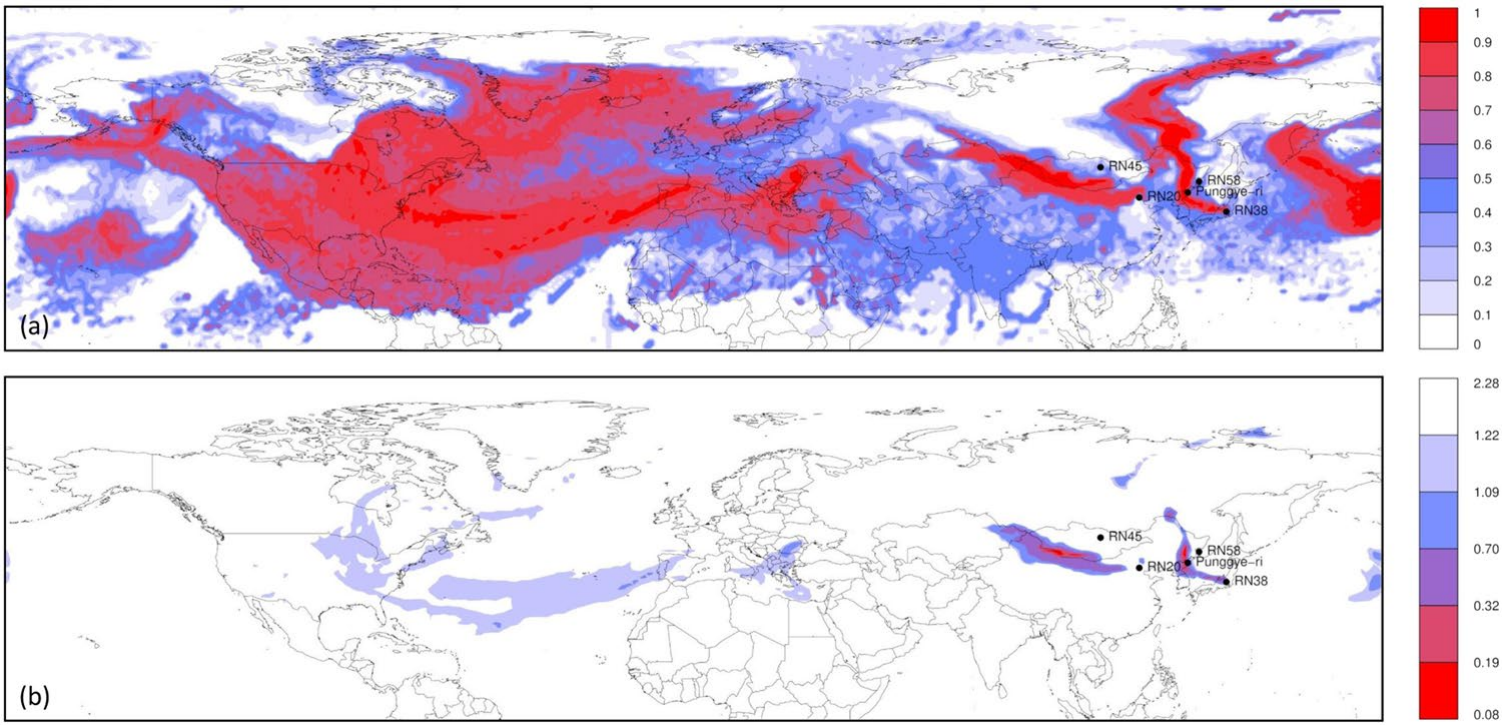
(**a**) Grid box maximum “Possible Source Region” (see text) for all simulation times for the northern hemisphere. (**b**) Minimum value of the cost function (Eq. 3 was used here) after inverse modelling was applied to each grid box separately. Maps were generated using the R statistical software^44,45^.

We started with assessing which areas in the northern hemisphere could be potential source locations, explaining the observations. The deterministic weather model was used with horizontal grid spacings of 1°. Two methods were used, the results of which are shown in Fig. 1. First, the correlation between the source-receptor-sensitivities and the observed ^133^Xe activity concentrations were calculated for all simulation times and for each grid box. This corresponds to the correlation between observed and simulated activity concentrations assuming a single short release (the duration of the release corresponds to the output frequency of the source-receptor-sensitivity fields by Flexpart, which was 3 hours). The result is what is sometimes called the “Possible Source Region” or PSR^15^. Since we were interested in the possible source locations and not in the release timing, the grid box maximum correlation was taken over the full simulation period of 26 days. The result is shown in Fig. 1a.

Second, inverse modelling using the optimisation technique described in Methods was used to obtain a cost function value for each grid box. The inverse modelling was applied once for the full simulation period, so that a single cost function value was obtained for each grid box, in contrast to the method where correlations were calculated.

The correlation map (Fig. 1a) shows a few large distinct areas in East Asia, as well as a huge area spanning from the Middle East to a large part of North America. Many grid boxes had a high correlation at some point in the simulation period, so that the correlation did not seem to be a good way to distinguish likely source regions from other regions, unless when using a very high correlation coefficient as threshold. The cost function values resulting from the inverse modelling are shown in Fig. 1b. Two distinct areas can be seen: the first lies roughly over the DPRK and the second lies near the border between China and Mongolia. These two regions have also a very high correlation (Fig. 1a).

When comparing both methods, it can be seen that the inverse modelling is better in discriminating likely source regions from other regions than the “Possible Source Regions” method: many grid boxes that had a high correlation, are unlikely according to the inverse modelling. One might wonder whether the difference could be attributed to the upper bound on the release term in the inverse modelling. However, the cost function maps obtained from applying inverse modelling with an upper bound increased by three orders of magnitude (*Q*_*max*_ = 10^16^ *Bq*/*day*) gave a roughly identical pattern (not shown).

### Focus on East Asia

**Figure 2:**
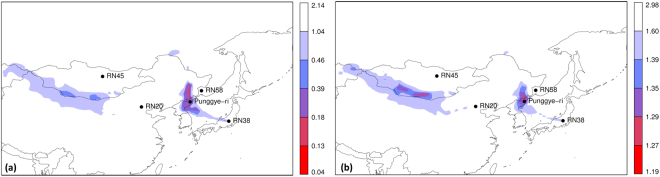
Grid box median cost function in the lowest model level using (**a**) Eq. 3 and (**b**) Eq. 4. The levels in the legend correspond to the 0, 0.0005, 0.001, 0.005, 0.01, 0.05 and 1 quantiles. Maps were generated using the R statistical software^44,45^.

Based on the assessment over the northern hemisphere, we identified two regions of interest located in East Asia. To assess these regions further, we used the unique data from the Ensemble Data Assimilation system of ECMWF. This resulted in a set of 51 ensemble members or meteorological scenarios representing the uncertainty in the meteorological data (Methods). For each ensemble member, we performed a set of backward Flexpart runs to calculate the source-receptor-sensitivity fields with horizontal grid spacings of 0.5°. Next, we performed inverse modelling (Methods) to obtain a set of 51 cost function values and optimal release terms for each grid box, one for each ensemble member. The inverse modelling was repeated twice, each time using one of the two cost functions described in Methods (Eqs 3 and 4). Figure 2 shows the resulting two maps of cost function values. The grid box median value was taken over all 51 cost function values. Both patterns of the cost function values are similar, so that the possible source locations are robust for the choice of the cost function.

**Figure 3:**
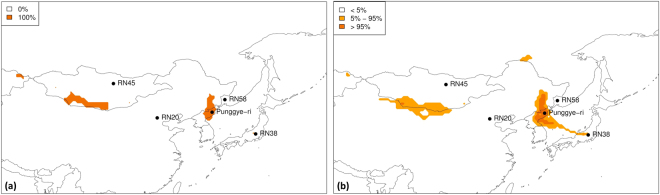
Grid box probability map using the cost function in Eq. 3 for (**a**) the unperturbed member only and (**b**) the full ensemble. Maps were generated using the R statistical software^44,45^.

In order to visualize the uncertainty captured by the ensemble of 51 cost function maps, a cost function threshold was first applied to discriminate possible source regions from other regions. The threshold was set at the 0.01 quantile of all grid box cost function values (the total number of grid boxes was 13,680). A possible source location probability map was then constructed by taking the percentage of members having a cost function below the threshold for each grid box, as was done in a previous study^19^. This is justified by the fact that in the Ensemble Data Assimilation system of ECMWF, each member can be considered equally likely, hence each member should have equal weighting. This is expected not to be the case for multimodel ensembles, where instead different weightings should be used depending on the member’s individual performance. The result for the inverse modelling using Eq. 3 is shown in Fig. 3. The left panel of Fig. 3 was obtained using the unperturbed meteorological data only (to better illustrate the added value of the ensemble versus the deterministic case), while the right panel was obtained using the full ensemble. The deterministic case shows two possible source regions (Fig. 3a). For the full ensemble, a discrimination was made between grid boxes for which at least 95% of the ensemble members agree that grid boxes are likely sources (dark orange area in Fig. 3b) or unlikely sources (white area in Fig. 3b). The likely sources regions were smaller when using the ensemble compared to the deterministic case, but a new region appeared where the ensemble members did not agree on whether a grid box was a likely source or not (yellow area in Fig. 3b). A notable difference between the deterministic and probabilistic case is the area near the Chinese-Mongolian border: the deterministic model showed that it is a likely source region, while the ensemble showed that it is associated with large uncertainties. The deterministic case can thus (i) be overly confident for certain grid boxes and (ii) perhaps worse, miss certain grid boxes that are likely source regions according to the ensemble.

## Influence of the ^133^Xe background

The radioxenon background varies significantly with time and space, and modelling its daily value at a specific location is not trivial. Furthermore, although significant effort has been put into understanding the global radioxenon background^21,22^, it could be that not all local sources of radioxenon are known. In this paragraph, we describe the assumptions that we made on the background, but we do not attempt to model it explicitly due to the uncertainties described here.

The signal of interest for which we wanted to find the possible source regions are the five samples that were shown in a previous study to be exceptional with respect to the station’s historic data due to the particular ratio of ^133^Xe and ^131*m*^Xe^14^. It was assumed that the signal of interest came from a single source (either a nuclear test or a civilian source). The background signal, defined as any other signal originating from one or multiple sources, was assumed to be significantly smaller than the signal of interest in the ^133^Xe samples containing more than 1 *mBq*/*m*^3^. In the other samples, having a ^133^Xe activity concentration ranging from below detection level up to a few tenths of *mBq*/*m*^3^, the contribution from the background was assumed to be anything up to 100%.

In this section, we assess the influence of the assumed ^133^Xe background on the inverse modelling.

### Robustness of the cost function to the background

We start by arguing that the cost functions used here (Eqs 3 and 4) have an inherent robustness against small background contamination of the samples. Indeed, both the mean square error and the correlation in Eq. 3 are resilient to small-scale perturbations on the radioxenon activity concentrations.

**Figure 4:**
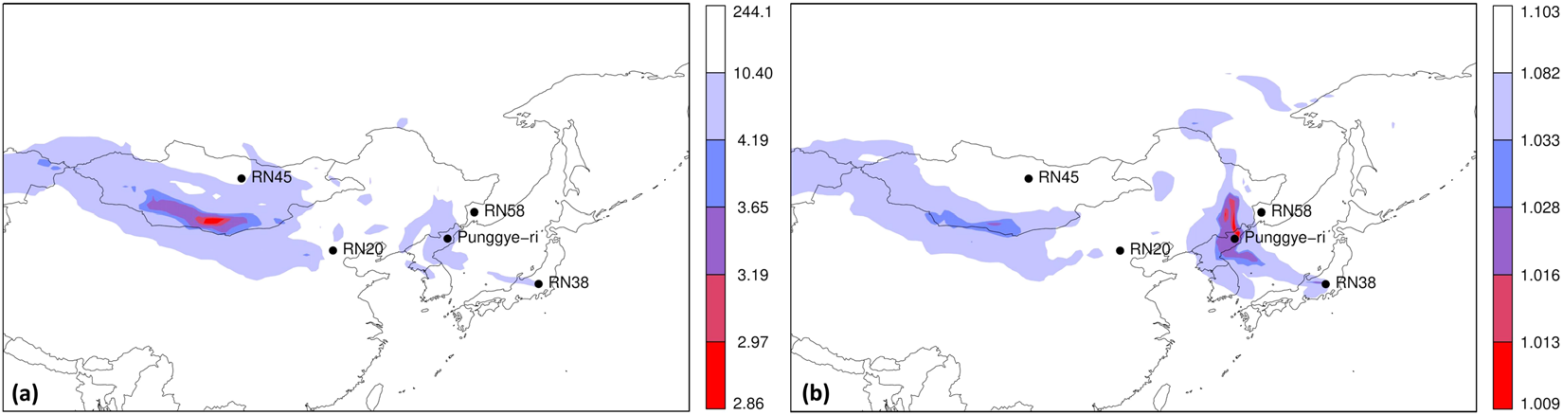
Grid box cost function in the lowest model level using Eq. 4 for different values for *α*: (**a**) *α* = 0.01 *mBq*/*m*^3^ and (**b**) *α* = 1 *mBq*/*m*^3^. The levels in the legend correspond to the 0, 0.0005, 0.001, 0.005, 0.01, 0.1 and 1 quantiles. Only the unperturbed member was used. Maps were generated using the R statistical software^44,45^.

The geometric variance treats all observations equally, but can have similar resilience via the parameter *α* in Eq. 4. This parameter *α* was added in the formula to allow the inclusion of non-detections in the inverse modelling (having activity concentrations ranging from zero to just below detection level). At the same time, it allowed for the adjustment of the tightness of fit between the observed and simulated activity concentrations. If a low value for *α* is chosen, observations with low activity concentrations should match equally well as observations with high activity concentrations. If a high value is attributed to *α*, the inverse modelling will be dominated by the information from observations with high activity concentrations. The value of *α* was set to 0.1 *mBq*/*m*^3^, slightly below the typical minimal detectable concentration. The effect of changing *α* in Eq. 4 on the source localisation is shown in Fig. 4. For very small *α* = 0.01 *mBq*/*m*^3^, and thus allowing a large influence of the background on the inverse modelling, the most likely source area is now found near the Chinese-Mongolian border. Although the area around the nuclear test site Punggye-ri is still distinguishable from other regions by a lower cost function, it is now a less likely source region (Fig. 4, left). When, on the other hand, a large *α* = 1 *mBq*/*m*^3^ was used, the inverse modelling now uses mostly information coming from samples with high activity concentration. The background will now have a small effect, with the risk of not using all relevant available information. The opposite effect can be seen on the cost function map: the area around the nuclear test site Punggye-ri is now the most likely source region (Fig. 4, right).

The value of *α* should be chosen based on the minimum detectable concentration and the typical background concentrations and is thus dependent on the tracer of interest. Its best value for ^133^Xe likely ranged from 0.1 *mBq*/*m*^3^ to 1 *mBq*/*m*^3^.

### Setting low ^133^Xe activity concentrations to zero

**Figure 5:**
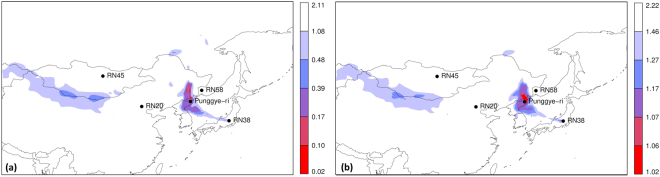
As Fig. 2, but with certain observations set to zero (see text) and for the unperturbed member only. As before, (**a**) was obtained using Eq. 3 and (**b**) using Eq. 4. Maps were generated using the R statistical software^44,45^.

The sensitivity to the background was further assessed by setting all ^133^Xe observations to zero, except for the five suspicious samples described in a previous study^14^ and for the elevated ^133^Xe measurement of 0.7 *mBq*/*m*^3^ taken at RN38 just after the three consecutive samples containing particular combinations of ^131*m*^Xe and ^133^Xe. The resulting cost function maps are shown in Fig. 5. It shows that the main features remain, which is not surprising since the cost functions are not very sensitive to low activity concentrations as discussed in the previous subsection. However, (i) the area near the Chinese-Mongolian border seems now a less likely source and (ii) the area of possible sources around DPRK is now more concentrated around the Punggye-ri nuclear test site.

### Inverse modelling using a subset of the observations

We now assess the degree of consistency among subsets of observations, meaning that all subsets of observations can be explained by one and the same single source. Due to the existence of the civilian radioxenon background, one might expect that observations will generally not be consistent. However, in the previous paragraphs we concluded that small inconsistencies do not significantly influence the inverse modelling source localisation. Here we selected 500 random subsets containing 75% of the observations, and applied inverse modelling on each of these subsets, using the unperturbed member only.

Such analysis could also be used to check for robustness against the case that a few samples contain wrong information, in the sense that the observed radioxenon is the signature of other sources than the source of interest. Indeed, using combinatorics, we can calculate the probability of not drawing *n* observations when a fraction *frac* = 75% of observations are drawn out of a total of *N* = 57 observations, and repeating this procedure *ntries* = 500 times:

1 $$p(n;\,N,\,frac,\,ntries)=1-{(1-\frac{(N-n)!(N-Nfrac)!}{N!(N-n-Nfrac)!})}^{ntries}$$

The probabilities for different values of *n* are given in Table 1. Note that with this setup, it is almost certain that any combination of three observations will not be selected at least once.

**Table 1:** Probability of not drawing *n* observations (*p*-values were calculated using Eq. 1).

*n*	*p*
1	≈1
2	≈1
3	0.998
4	0.72
5	0.21
6	0.04

**Figure 6:**
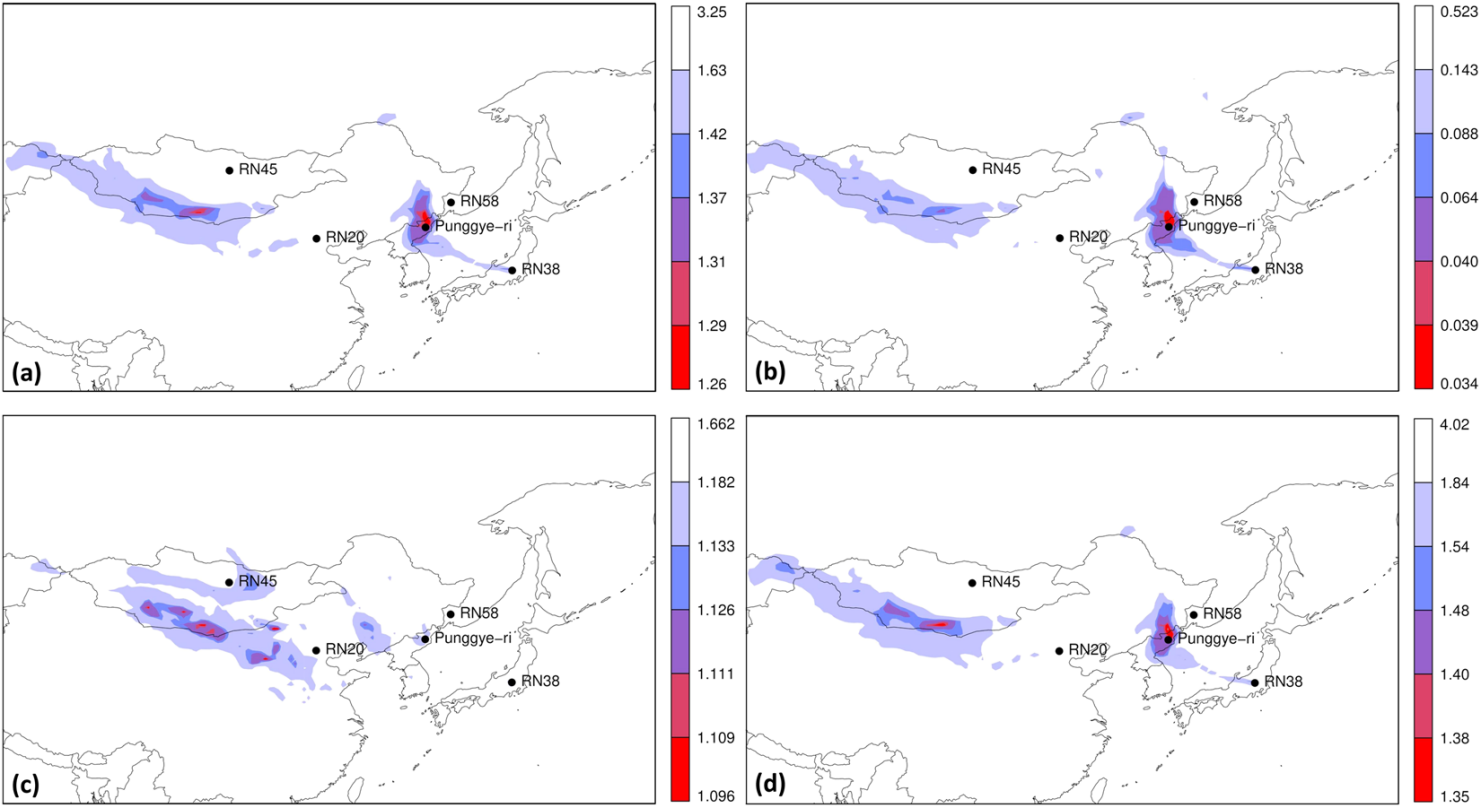
From 500 cost function maps obtained from different subsets of observation, the following statistics are plotted: (**a**) grid box median of the cost function; (**b**) grid box standard deviation of the cost function; (**c**) grid box minimum cost function; (**d**) grid box maximum cost function. The levels in the legend correspond to the 0, 0.0005, 0.001, 0.005, 0.01, 0.05 and 1 quantiles. Maps were generated using the R statistical software^44,45^.

For each of the 500 subsets of observations, inverse modelling was applied. Equation 4 was used, since both cost functions gave similar results and the inverse modelling was slightly faster compared to Eq. 3. In order to interpret the resulting 500 cost function maps, we plotted the grid point minimum, maximum, median and standard deviation of the cost function value in Fig. 6 (as such, it could well be that in Fig. 6, different grid boxes make use of different subsets of observations). The grid box median of the cost function (Fig. 6a) is very similar to the calculation using all observations (Fig. 2b). This confirms indeed that the inverse modelling was consistent for subsets of observations, and that small perturbations from the background did not strongly influence the results. When plotting the grid box standard deviation of the cost function (Fig. 6b), the two possible source regions are shown to be robust for selecting different subsets of observations, especially the region around the Punggye-ri test site. The grid box minimum and maximum cost function values were also plotted (Fig. 6c and d). Figure 6c shows that it is possible to find a combination of observations for which a pattern was found significantly differing from previous patterns. However, this pattern corresponded to the case where the largest three ^133^Xe observations were omitted from the inverse modelling, while it was assumed that these contain most information on the event of interest. As such, it should not surprise that a different pattern was found. When requiring that a grid box should comply with all subsets of observations, which can be obtained by taking the grid box maximum of the cost functions (Fig. 6d), we found a pattern roughly identical to the median (Fig. 6a).

### Source localisation of the Xe-131m detections

**Figure 7:**
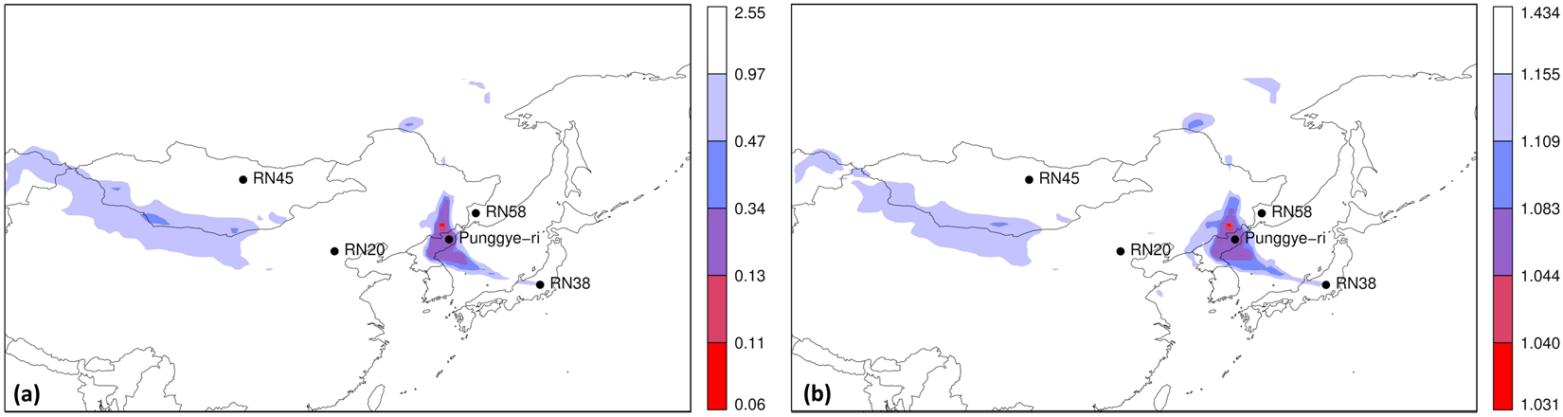
As Fig. 2, but for the ^131*m*^Xe observations. Maps were generated using the R statistical software^44,45^.

The inverse modelling was repeated using 57 ^131*m*^Xe observations (the same period and stations as for the ^133^Xe observations as described in Methods were used). The cost function map is shown in Fig. 7. Not surprisingly, a pattern similar to Fig. 2 can be observed. Indeed, the same meteorological data were used, consequently the source-receptor-sensitivities are similar (the only difference comes from the radioactive decay since ^131*m*^Xe and ^133^Xe have different half-lives). Furthermore, the observations of ^131*m*^Xe and ^133^Xe are strongly correlated: the Pearson correlation coefficient is 0.86 for these 57 observations. Figures 2 and 7 differ noteworthy in the area around the Punggye-ri nuclear test site, which is the single most likely source region in Fig. 7, for both cost functions Eqs 3 and 4.

### Hypothetical radioxenon release profile at the Punggye-ri nuclear test site

**Figure 8:**
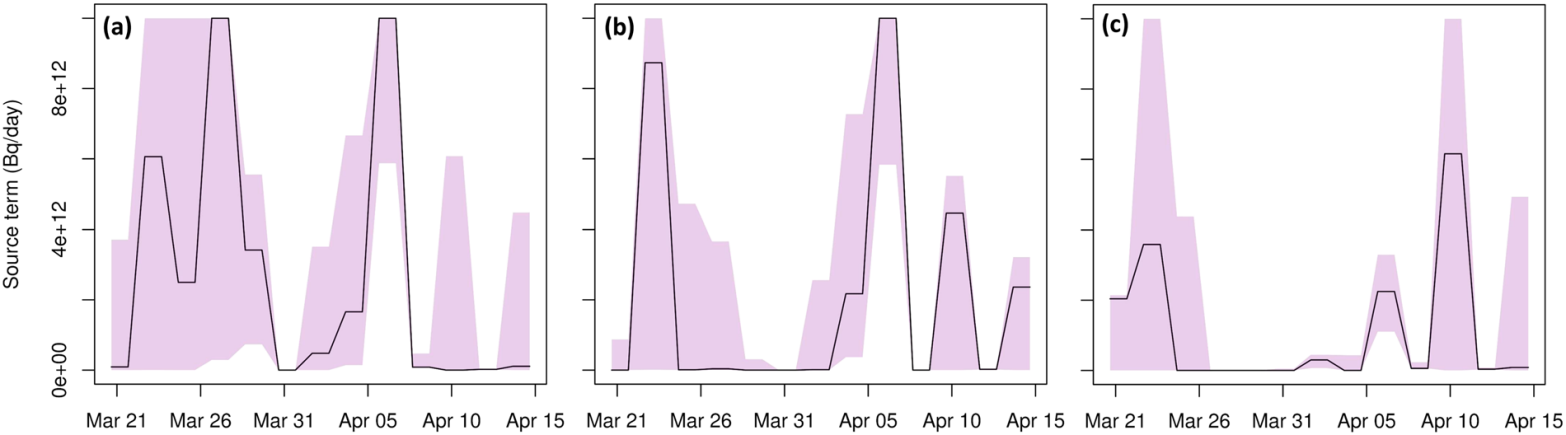
Release profile (black solid line) obtained from the unperturbed member for the Punggye-ri nuclear test site: (**a**) ^133^Xe release profile using unadapted ^133^Xe observations, (**b**) ^133^Xe release profile using modified ^133^Xe observations and (**c**) ^131*m*^Xe release profile using unchanged ^131*m*^Xe observations. The shadings represent the 0.025 and 0.975 quantiles of the full ensemble. The figure was made using the R statistical software^44^.

In this section, it is assumed that the radioxenon signal of interest originated from a delayed release at the Punggye-ri nuclear test site. The corresponding radioxenon release profile was calculated for three sets of observations: (i) unmodified ^133^Xe observations, (ii) the ^133^Xe observations changed as discussed previously (where most ^133^Xe observations were set to zero) and (iii) unmodified ^131*m*^Xe observations. The resulting three release profiles are shown in Fig. 8. The full ensemble was used, from which uncertainty bounds were created. Eq. 3 was used as cost function, though similar features were found when using Eq. 4 (not shown).

The release profile obtained using unmodified observations (Fig. 8a) shows a possible release between 21 and 30 March 2013, but the associated uncertainties are very large. Around 27 and 29 March, most ensemble members predicted a significant release, although the exact amount released showed quite some spread among the ensemble members. Between 6 and 7 April, a sharp release was found with less uncertainty. The ensemble revealed that the release could have started a few days earlier. Finally, the ensemble also showed the possibility of releases around 10 April and 14 April but with a large degree of uncertainty.

When using modified observations as discussed previously with the aim to minimise the effect of the background, we see that the releases between 26 and 30 March 2013 had decreased significantly. The uncertainty bounds obtained from the ensemble suggests that the only significant release occurred between 4 and 7 April. The additional release in Fig. 8(a) compared to Fig. 8(b) is the result of the inverse modelling trying to fit the simulated activity concentrations with the observed low activity concentrations. However, due to the construction of the cost function, these have a small impact on the cost function. As a result, large differences in the release term can lead to only small differences in the cost function. The additional release should therefore not be interpreted too strictly, and the release profile obtained from the modified observations, showing a single robust release, is by far the most interesting feature. Note that the uncertainty bounds from the ensemble are helpful for carefully interpreting the additional releases.

The ^131*m*^Xe release profile is shown in Fig. 8(c). As for the ^133^Xe release profile, a significant release is predicted around 6–7 April. Note that this release is lower than for the ^133^Xe release profile. This is expected since at the time of the potential release, the ^131*m*^Xe inventory is still smaller than the ^133^Xe inventory due to the different cumulative fission yields^23^.

### Summary and conclusions

Several weeks after the third announced nuclear test conducted by the Democratic People’s Republic of Korea, particular combinations of ^131*m*^Xe and ^133^Xe had been measured at two noble gas stations that are part of the International Monitoring System to verify compliance with the Comprehensive Nuclear-Test-Ban Treaty^14^. In this paper, the origin of this radioactive xenon has been assessed using long-range atmospheric transport and dispersion modelling. Unique meteorological ensemble data were obtained by running the latest version of the Ensemble Data Assimilation system of ECMWF for the period March-April 2013. Atmospheric transport and dispersion calculations were performed with Flexpart in backward mode to calculate the source-receptor sensitivity fields for 57 noble gas observations from the International Monitoring System. Inverse modelling was used to search for an optimal source term in each grid box of the domain that matched best with the observations (“best” being quantified by two alternative cost functions Eqs 3 and 4). Using the adjoint approach, there was no need to rerun the atmospheric transport model during the optimisation. The ensemble allowed to quantify the source location uncertainty and release uncertainty.

The source localisation of the ^133^Xe detections showed two distinct possible source regions: one at the Chinese-Mongolian border, and one around the Punggye-ri nuclear test site (Fig. 2). The results were robust for the two alternative cost functions that were used in this study. The correlation between the source-receptor-sensitivity fields and the observed activity concentrations (sometimes called the “Possible Source Regions” product) turned out to be poor in discriminating possible source regions from other regions when the timing of the release is not specified (Fig. 1). The ensemble allowed to construct grid-pointwise probability maps such as Fig. 3. This provided additional valuable information compared to the deterministic result.

A particular challenge for the radioxenon verification part of the CTBT is the existence of a radioxenon background from civilian sources. The influence of the ^133^Xe background on the source location was assessed indirectly. The assessment suggests that the possible source region around the Chinese-Mongolian border should at least partly be attributed to the ^133^Xe background (partly, since the specific meteorology for that period and the noble gas station network configuration also influence the size of possible source regions). This was observed when (i) more weight was given to the observations having a high ^133^Xe activity concentration (Fig. 4b) and (ii) certain detections were changed to non-detections (Fig. 5). Furthermore, the results were consistent among different subsets of observations, such that the findings are not negatively influenced by a few samples containing ^133^Xe from sources other than the source of interest. This discussion highlights the importance of having a thorough understanding of the background, so that sound assumptions can be made when assessing the influence of the background. The parameter *α* in Eq. 4 was kept constant in this study, but it can readily be extended to be station-specific or even sample-specific (representing the fact that different noble gas stations have different radioxenon backgrounds or samples have different minimum detectable concentrations).

The source localisation was repeated for the ^131*m*^Xe detections. The resulting possible source locations were similar to those for ^133^Xe. However, the area around the Punggye-ri nuclear test site was now the single most likely source region (Fig. 7).

When a delayed ^133^Xe release from the Punggye-ri nuclear test site is assumed, a sharp release around 6–7 April 2013 is observed (Fig. 8). The additional releases with large uncertainties according to the ensemble were partly removed by using the modified observations to minimise the effect of the background.

Currently, all four radioxenon isotopes are rarely (if at all) measured in a single sample by the International Monitoring System, making a routine discrimination of samples based on the discrimination line^24^ not feasible. However, this technique might become useful in the future since the noble gas detection systems are further being improved, making it more likely that all four radioxenon isotopes will be measured simultaneously^25,26^. To date, the detection of radioxenon combined with atmospheric transport modelling provides most feasibly evidence to confirm the nuclear nature of a man-made explosion. The source localisation of both ^131*m*^Xe and ^133^Xe indicate that a delayed release from the Punggye-ri nuclear test can explain the observations, and the inverse modelling greatly confines possible source regions.

## Methods

### Radioxenon observations

Radioxenon detections and non-detections made by the International Monitoring System were used for the inverse modelling. In order to confine the possible source regions of the radioxenon detections as much as possible, many observations should be used, since each detection or non-detection carries some information. However, detections can be contaminated by the radioxenon background from civilian emitters. It is challenging to estimate the correct radioxenon contribution from civilian emitters for every individual sample^27,28^ (especially if not all civilian sources of radioxenon in the region are known). This highlights the importance of understanding the radioxenon background and reducing radioxenon emissions from civilian sources^29^. With the above considerations in mind, we selected all ^133^Xe observations taken between 5 April 2013 and 15 April 2013 for the IMS noble gas stations RN20, RN38, RN45 and RN58 (see Fig. 2 for their location). In total, 57 observations were used for the inverse modelling (Supplementary Information).

The IMS noble gas stations RN20 and RN45 are equipped with the Spalax system^30,31^ and measure radioxenon activity concentrations every 24 h, whereas RN38 is equipped with the Sauna system^32^ and measures radioxenon activity concentrations every 12 h. Finally, RN58 is equipped with the Arix system and measures radioxenon activity concentrations every 12 h^33^.

### ATM model and weather data

Two sets of meteorological data were used. First, deterministic weather data of the Integrated Forecasting System of the European Centre for Medium-Range Weather Forecasts (ECMWF) (4 analyses per day were combined with forecasts having a 3 h lead time, so that meteorological data were available every 3 h). Second, unique meteorological data created by running the current latest version (cy43r1) of ECMWF’s Ensemble Data Assimilation (EDA) system^34^ for the period March-April 2013. This ensemble consisted of 26 independent lower-resolution 4D-Var assimilations, of which 25 use perturbed observations, sea-surface temperatures and model physics. More information on the type of perturbations can be found in another study^34^. The EDA cycles its own background error and covariance estimates, therefore it can be seen as a variational implementation of a perturbed observation Ensemble Kalman Filter. For each perturbed EDA member, the perturbations with respect to the ensemble mean were calculated. These perturbations were then added and subtracted from the unperturbed member, so that 50 perturbed members were obtained. Meteorological data were available every three hours, having horizontal grid spacings of 0.5° and having 137 non-uniform vertical levels with the top level at 0.01 hPa.

The Lagrangian particle model Flexpart^35–37^ version 9.02 was used for the atmospheric transport and dispersion calculations. Flexpart was used in backward mode^38^, giving source-receptor-sensitivities as output. Radioactive decay was taken into account for both ^133^Xe and ^131*m*^Xe. The ECMWF Mars extraction software for Flexpart available at the Flexpart website^39^ was used in modified form.

### Inverse modelling method

Consider a vector of observed concentrations **y**. Inverse modelling involves finding a source term **x**(*x*, *y*, *z*, *t*) so that the following relation is true:

2 $${\bf{y}}={\bf{M}}\,{\bf{x}}+\varepsilon $$

Here, **M** is the source-receptor-sensitivity matrix^38,40^ and *ε* is the combined observation and model error. Forward modelling is often used to calculate **Mx** after selecting an initial guess source term **x**. Via an iterative process, the source term is then refined. However, with Flexpart, it is possible (and, if the source location is not known, more efficient) to perform a backward calculation for each observation used in the inverse modelling; the result is the source-receptor-sensitivity matrix **M**, thereby avoiding the need to rerun the atmospheric transport model during the optimisation: only the source term **x**(*x*, *y*, *z*, *t*) must be varied until Eq. 2 holds.

An exact match between simulations and observations is not possible since both the source-receptor-sensitivity matrix and the observations contain uncertainties. Instead, the disagreement between the observed activity concentrations ***y*** and the simulated activity concentrations **Mx** should be minimised while taking into account the uncertainty in the observations and the model. The disagreement is quantified by a cost function. Two cost functions were used in this study. A previous study^41^ combined the mean square error (*mse*) and the correlation (*cor*) into a single likelihood weight. It was argued that *mse* is mainly sensitive to changes in source amount, while temporal *cor* is mainly sensitive to changes in arrival time and duration. We followed this reasoning for constructing the following cost function (note the normalisation of the *mse*):

3 $${\rm{cost}}\,{\rm{function}}({\bf{x}})=\sum _{samples}{(A{C}_{obs}-A{C}_{sim})}^{2}\cdot {(\sum _{samples}{(A{C}_{obs})}^{2})}^{-1}+1-cor(A{C}_{obs},A{C}_{sim})$$

where *AC*_*obs*_ and *AC*_*sim*_ are the observed and simulated activity concentration. An alternative cost function used in this study is the geometric variance (Eq. 4). This cost function was found to give the best results when applying inverse modelling to a large domain and noisy measurements^42^. Here, a parameter *α* has been added representing the minimum detectable concentration, such that non-detections could be used in the inverse modelling:

4 $${\rm{cost}}\,{\rm{function}}({\bf{x}})=\exp (\frac{1}{n}\,\sum _{samples}{(\mathrm{log}(A{C}_{obs}+\alpha )-\mathrm{log}(A{C}_{sim}+\alpha ))}^{2})$$

with *n* = 57 the number of observations and log the natural logarithm. We assumed that the signal of interest originated from a single grid box source. Therefore, for each grid box in the lowest model level, we performed the inverse modelling and obtained a cost function value and an associated optimal source term. It was assumed that the most likely source locations are those grid boxes with the lowest cost function values.

The release is assumed to took place between 20 March 2013 and 15 April 2013, with release intervals of two days. The latter is sufficient to resolve the synoptic scale signal^43^ and helps to regularise the problem. Although such a long release window complicates the inversion due to longer computation times and more unknowns in the inversion, it allows to identify remote sources.

The optimisation was done using the routine *nlminb* from the R statistical software^44^ that uses a quasi-Newton method for the minimisation with bounds on the release term (*Q*_*min*_ = 10^9^ Bq/day and *Q*_*max*_ = 10^13^ Bq/day were used unless otherwise mentioned).

### Data availability

The data generated during and/or analysed during the current study are available from the corresponding author on reasonable request.

## Electronic supplementary material

Supplementary Info Radioxenon observations
