# Supplementary material for: Source localisation and its uncertainty quantification after the third DPRK nuclear test
Source: Sci Rep. 2018 Jul 5;8:10155. doi: 10.1038/s41598-018-28403-z (PMC6033904; doi:10.1038/s41598-018-28403-z)
Supplement: Supplementary file 1 — Supplementary Info Radioxenon observations [file 41598_2018_28403_MOESM1_ESM.pdf]

Supplementary Information to "Source localisation and its uncertainty quantification after the third DPRK nuclear test"

Pieter De Meutter[1,2,3], Johan Camps[1], Andy Delcloo[2,3], Piet Termonia[2,3]

[1]: Belgian Nuclear Research Institute, Mol, 2400, Belgium

[2]: Royal Meteorological Institute of Belgium, Brussels, 1180, Belgium

[3]: Ghent University, Department of Physics and Astronomy, Ghent, 9000, Belgium

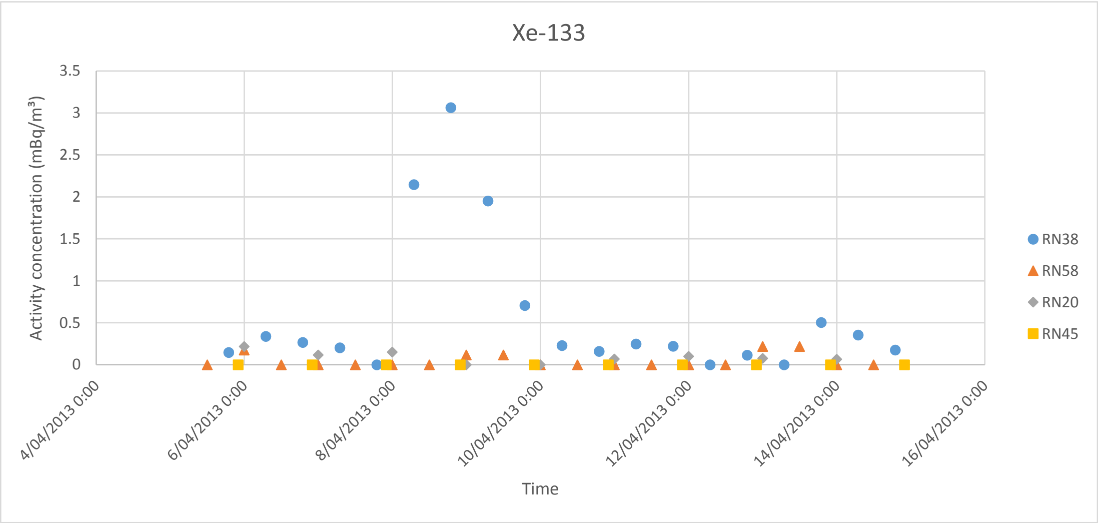

Figure 1: Time series (in UTC) of the observed activity concentrations of Xe-133 that were used in the inverse modelling.

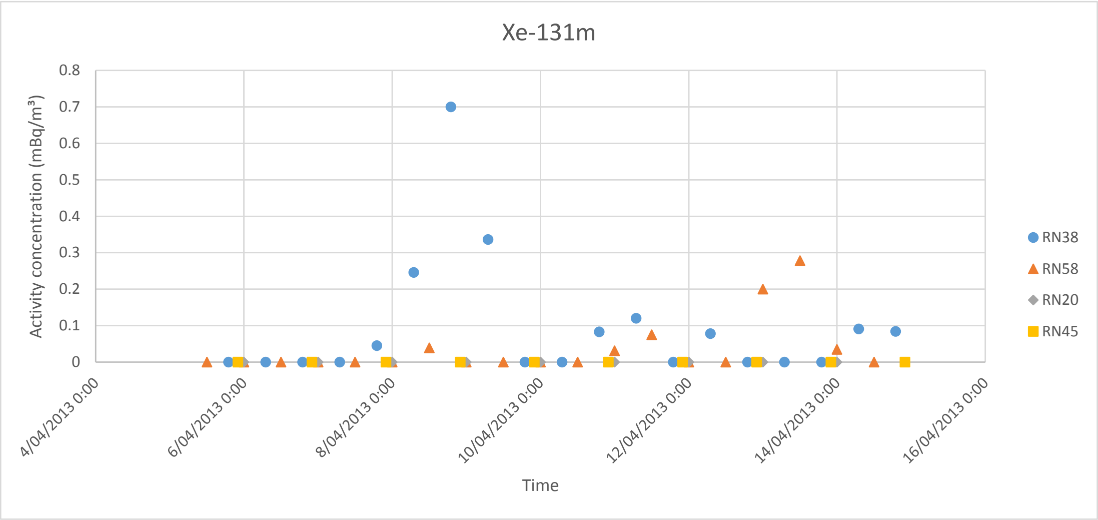

Figure 2: Time series (in UTC) of the observed activity concentrations of Xe-131m that were used in the inverse modelling.
